# Efficacy and safety of the tree sublingual immunotherapy tablet in the subpopulation of Canadian children with allergic rhinitis and/or conjunctivitis: phase III trial results

**DOI:** 10.1186/s13223-026-01054-w

**Published:** 2026-07-11

**Authors:** Rémi Gagnon, Terrie Dalgaard, Gry Schermann Ortving, Monika Gappa, Hendrik Nolte

**Affiliations:** 1 Clinique Spécialisée en Allergie de la Capitale, 2600 Boul. Laurier suite 880, Québec, QC G1V4W2 Canada; 2 ALK, Hørsholm, Denmark; 3 Evangelical Hospital Düsseldorf, Düsseldorf, Germany; 4 ALK, Bedminster, NJ USA

**Keywords:** Allergic rhinitis, Birch, Pollen, Sublingual immunotherapy, Tree, Oak, Tablet

## Abstract

**Background:**

Pollen from birch and other trees in the birch homologous group (e.g., oak) is a common trigger of allergic rhinitis and/or conjunctivitis (AR/C) in Canada. The efficacy and safety of the tree sublingual immunotherapy (SLIT)-tablet in children with AR/C was evaluated post hoc in the Canadian subpopulation of a phase III, double-blind trial (EudraCT2020-004372-17).

**Methods:**

Children and adolescents aged 5–17 years with moderate-to-severe tree AR/C were randomized to either daily tree SLIT-tablet or placebo for up to 52 weeks. Participants had free access to symptom-relieving medication. The primary endpoint was the average total combined score (TCS; sum of rhinoconjunctivitis daily symptom score [DSS] and daily medication score [DMS]) during birch pollen season (BPS). Average TCS during tree pollen season (TPS, consisting of birch, alder, hazel, and oak pollen seasons), oak pollen season (OPS), and pure OPS (excluding days from birch, alder, or hazel pollen seasons) were also analyzed.

**Results:**

In the Canadian subpopulation (*n* = 87), treatment with the tree SLIT-tablet reduced the average TCS by 26.8% versus placebo during BPS (absolute difference [95% CI] = 1.89 [-0.71, 4.49], *p* = 0.138), 32.0% during TPS (absolute difference = 2.14 [-0.03, 4.32], *p* = 0.042), 46.6% during OPS (absolute difference = 3.70 [0.98, 6.42], *p* = 0.004), and 50.6% during pure OPS (absolute difference = 4.20 [1.12, 7.27], *p* = 0.005). Reductions in DSS and DMS with tree SLIT-tablet versus placebo were also observed.

In the total trial population (*N* = 952), tree SLIT-tablet treatment reduced the average TCS by 19.2% versus placebo during BPS (absolute difference = 1.13 [0.42, 1.84], *p* = 0.002), 16.8% during TPS (absolute difference = 0.76 [0.26, 1.26], *p* = 0.003), 23.4% during OPS (absolute difference = 1.32 [0.60, 2.05], *p* < 0.001), and 19.9% during pure OPS (absolute difference = 1.03 [0.27, 1.80], *p* = 0.009). The tree SLIT-tablet was well tolerated in the total trial and Canadian subgroup populations.

**Conclusions:**

The tree SLIT-tablet demonstrated favorable efficacy and was well tolerated in Canadian children and adolescents with AR/C.

**Clinical trial registration:**

EudraCT 2020-004372-17 and NCT04878354.

**Supplementary Information:**

The online version contains supplementary material available at https://doi.org/10.1186/s13223-026-01054-w.

## Background

Tree pollen is a common trigger of allergic rhinitis and/or conjunctivitis (AR/C) in Canada [1–3]. Birch and oak trees are found throughout Canada [4, 5], and depending on the region, sensitivity to birch occurs in approximately 25–35% of atopic individuals [6–8]. The prevalence of sensitivity to oak in Canada has not been well-documented; however, antibodies to birch cross-react with oak [9], and exposure to oak pollen can trigger AR/C symptoms in individuals sensitized to birch and vice versa [10, 11]. 

Symptoms of AR/C can have a profound negative impact on patients. In the Allergies in Canada survey of 1,001 patients with allergic rhinitis, 72% reported that their symptoms adversely affected their daily lives during allergy season, and nearly 1 in 4 patients (23%) reported reduced productivity [12]. In the Canadian Allergy Patient Identification for Immunotherapy (AsPIRe) survey of 136 patients with seasonal allergic rhinitis, 40% reported being affected by their allergy symptoms 6–7 days a week, and > 60% reported being affected 4 or more days per week [13]. In addition, peak birch and oak pollen seasons correlate with increased AR/C symptom-relieving medication use and asthma-related hospitalization and emergency department visits [14–16]. 

Allergy immunotherapy is a disease-modifying treatment option for AR/C [17]. Sublingual immunotherapy (SLIT) tablets are approved in Canada for children ages 5 years and older who have AR/C induced by tree, ragweed, grass, and house dust mite allergens. The favorable safety profile versus subcutaneous immunotherapy, the ability to administer at home, and the avoidance of injections may make SLIT-tablets an appealing allergy immunotherapy option for children and their caregivers [18]. Findings from a pivotal phase III, double-blind, randomized, placebo-controlled trial evaluating the efficacy and safety of the tree SLIT-tablet in children and adolescents with AR/C in Europe, Russia, and Canada were instrumental in the subsequent approval of the tree SLIT-tablet for patients over the age of 5 years in Canada [19]. 

Biomes differ among the US, Europe, and Canada, which could subsequently affect the distribution of birch and cross-reactive tree species (e.g., oak, beech, alder, hazel) and impact the efficacy of the tree SLIT-tablet. The pollen-induced symptom severity is patient-dependent (e.g., potential preseasonal priming of the immune response by exposure to other allergens for polysensitized patients) and determined by the amount of pollen at the time of exposure [20]. Although the preseasonal priming or the amount of pollen may differ among biomes, the amount has no effect on the mechanism of the IgE-mediated response itself, but it may impact the observed magnitude of the treatment effect. While allergen sensitization profiles and allergy immunotherapy practices differ across global regions [21, 22], subgroup analyses of grass and ragweed SLIT-tablet trials conducted in North America, or North America and Europe, have demonstrated consistent efficacy and safety between the Canadian participants and the full trial populations [23–25]. In light of this, the current post hoc analysis evaluated whether a subgroup of Canadian children with AR/C who participated in the phase III tree SLIT-tablet trial would also exhibit efficacy and safety outcomes comparable with those observed in the overall trial population.

## Methods

### Trial design

This was a phase III, randomized, double-blind, placebo-controlled trial conducted in Europe, Canada, and Russia (EudraCT 2020-004372-17 and NCT04878354). The study design, inclusion and exclusion criteria, randomization procedures, endpoints, statistical methods, and primary results have been previously described in detail [19]. Briefly, participants were randomized to receive daily placebo or SQ tree SLIT-tablet (12 SQ-Bet dose containing standardized allergen extract from birch pollen; ALK-Abello, Hørsholm, Denmark) starting at least 12 weeks before the anticipated tree pollen season (TPS) and continuing through the TPS, corresponding to a total treatment duration of up to 52 weeks (**Supplemental Figure ****S1**). The first dose was administered under clinical observation at the study site. Participants were allowed to use open-label symptom-relieving medication provided by the study sponsor throughout the TPS.

There were two separate trial enrollment cohorts over two consecutive years (cohort 1 = 2022 TPS; cohort 2 = 2023 TPS). Eligible participants were individuals aged 5–17 years with moderate-to-severe birch pollen AR/C despite the use of symptom-relieving medication for at least one TPS, with or without a medical history of asthma. Participants had to have a positive skin prick test (wheal size ≥ 3 mm) and specific IgE (≥ 0.70 kU/L) to *Betula verrucosa* at screening, as well as the presence of at least one of the following Allergic Rhinitis Impact on Asthma quality of life items due to AR/C during the previous birch pollen season (BPS): sleep disturbance; impairment of daily activities, leisure, and/or sport; impairment of school or work; troublesome symptoms.

Written informed consent was obtained from each participant’s legal guardian, and the trial was conducted in accordance with the Declaration of Helsinki and Good Clinical Practice. The trial protocol was reviewed and approved by each study sites’ institutional review board.

### Study assessments

Participants or caregivers recorded symptoms and symptom-relieving medication use daily in an eDiary during the TPS. The primary endpoint was the average total combined score (TCS) during the BPS. The TCS is the sum of the rhinoconjunctivitis daily symptom score (DSS) and the rhinoconjunctivitis daily medication score (DMS). Key secondary endpoints were the average TCS during TPS, and average DSS and DMS during BPS and TPS. The average TCS, DSS, and DMS during oak pollen season (OPS) were secondary endpoints, and the average TCS, DSS, and DMS during pure OPS were assessed post hoc. Safety assessments of adverse events (AEs) and events of special interest were collected throughout the trial.

Tree pollen counts were measured in Quebec and Montreal. The BPS in each region was defined as beginning on the first day of 3 consecutive days with a birch pollen count of ≥ 30 grains/m^3^ and ending on the last day in the last occurrence of 3 consecutive days with a birch pollen count of ≥ 30 grains/m^3^ [26–29]. The same definition applied for hazel, alder, and oak pollen seasons but with a threshold of ≥ 10 grains/m^3^. The TPS was defined as all days falling within any birch, hazel, alder, or oak pollen season (not necessarily a continuous period). Pure OPS was defined as the days in the OPS with no overlap of the birch, alder, or hazel pollen seasons.

Rhinitis symptoms of runny nose, blocked nose, sneezing, and itchy nose and conjunctivitis symptoms of gritty feeling/red/itchy eyes and watery eyes were scored on a scale of 0–3 (none, mild, moderate, severe), for a maximum rhinoconjunctivitis DSS of 18. Use of symptom-relieving medications (oral antihistamines, ocular antihistamines, and intranasal corticosteroids) were scored as previously described [19], for a maximum rhinoconjunctivitis DMS of 20. Thus, the maximum TCS was 38.

### Statistical analysis

Endpoints in the Canadian subpopulation of the trial were analyzed post hoc using the treatment policy estimand, a conservative statistical approach recommended by the International Council for Harmonization guidance for clinical trials [30]. Endpoints in the full trial population were analyzed using the treatment policy estimand a priori for the BPS and TPS and post hoc for the OPS and pure OPS. The treatment policy estimand approach uses all collected data, including data collected after a participant discontinued treatment, to reflect treatment effectiveness under real-world conditions. Missing values due to lack of efficacy or treatment-related AEs were imputed from placebo, and missing values due to other reasons were imputed from the participant’s own treatment group.

Imputation was done within age strata using the method of unrestricted random sampling with replacement and 1000 multiple imputed datasets were created. Each of the 1000 multiple imputed datasets was analyzed using the square root transformed endpoint as the response variable in an linear mixed effects model which included treatment, age group, and cohort as fixed factors, and pollen station within cohort as a random effect, with different residual errors specified for each treatment group, and with denominator degrees of freedom calculated using Kenward and Roger’s approximation. Rubin’s rule was used to combine the analysis results across the multiple imputed datasets. The results were back-transformed from the linear mixed effects model, estimated least square means on the square root transformed scale were output along with associated covariance matrix. For the absolute difference, the standard error was approximated by using the first order Delta method (first order Taylor approximation), and from this the 95% CI was calculated. For the relative difference between treatment groups, Fieller’s theorem was first used to calculate the 95% CI, and then this was back-transformed by applying a monotone transformation.

## Results

**Fig. 1:**
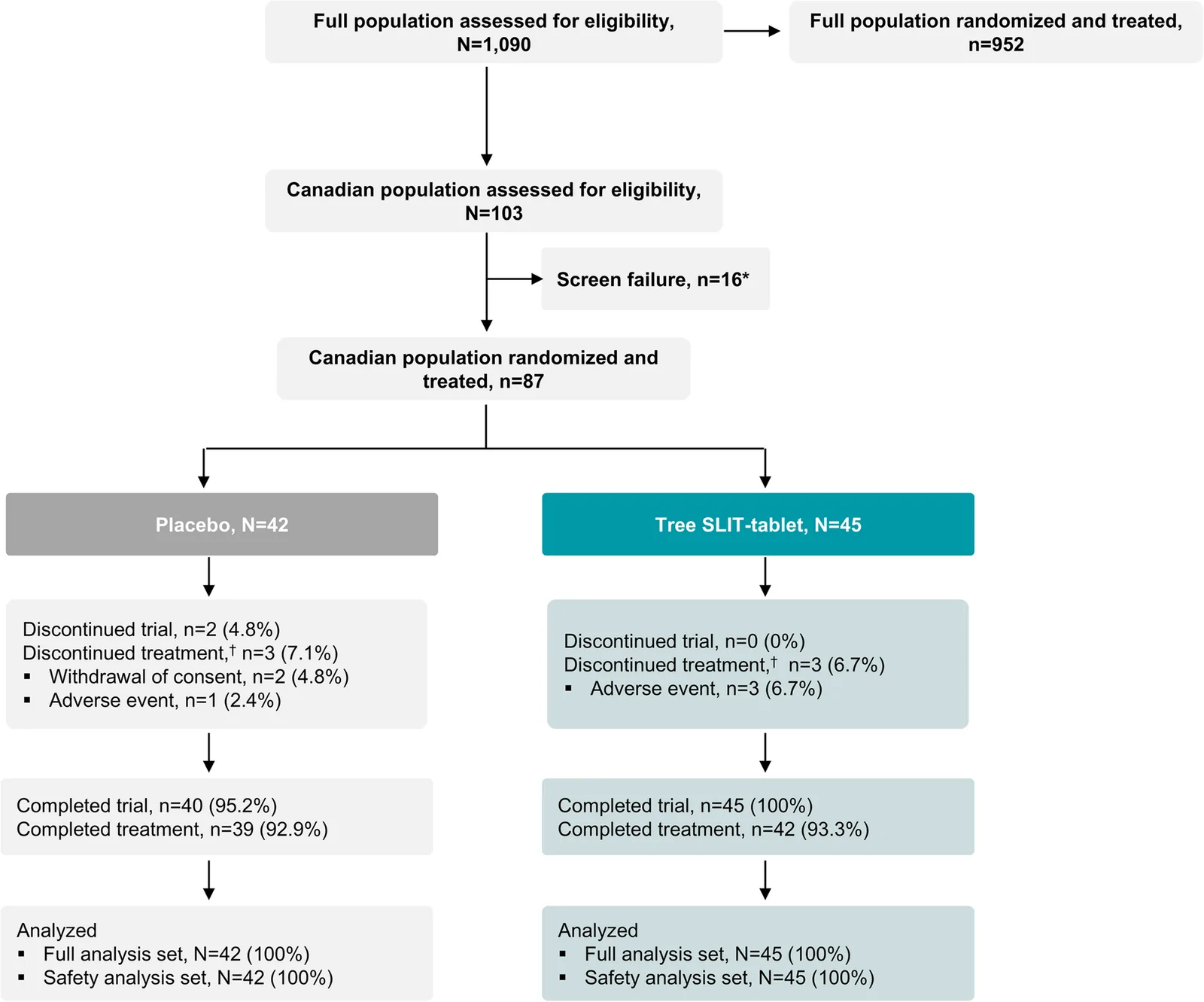
Canadian participants disposition. All participants were sensitized to birch pollen, except one participant in the placebo group who was randomized in error. This participant was included in the trial due to positive IgE results but was excluded from the supplementary analysis. ^*^Reason for screen failure: inclusion criteria required a positive IgE and skin prick test to *Betula verrucosa* at screening; ^†^Discontinued treatment was based on the end of the study electronic case report form. SLIT, sublingual immunotherapy

*Participants* Overall, 952 participants were randomized and treated, 87 of whom were enrolled from Canada (Fig. 1). The mean age of the Canadian subpopulation was 10.8 years, and nearly all participants (96.6%) were White (Table 1). There were twice the number of males than females in the tree SLIT-tablet group (Table 1). The majority (83.9%) of participants were polysensitized and 26.4% had asthma (Table 1).

**Table 1:** Demographics and baseline characteristics of the Canadian subpopulation

	Placebo (*N* = 42)	Tree SLIT-tablet (*N* = 45)
Age, y, mean (SD)	10.4 (3.1)	11.2 (2.9)
Age by group, n (%)		
5–11 y	23 (54.8)	25 (55.6)
12–17 y	19 (45.2)	20 (44.4)
Male, n (%)	24 (57.1)	30 (66.7)
Race, n (%)		
White	42 (100)	42 (93.3)
Black or African American	0	1 (2.2)
Multiple	0	2 (4.4)
Not Hispanic or Latino	42 (100)	44 (97.8)
Baseline sensitisation based on SPT, n (%)		
Birch only	9 (21.4)	5 (11.1)
Birch and others	33 (78.6)	40 (88.9)
Asthma at baseline, n (%)	9 (21.4)	14 (31.1)
Baseline specific IgE, mean kU/L (SD)		
Birch	67.2 (34.0)	57.2 (37.4)
Alder	46.6 (44.3)	44.0 (48.8)
Hazel	28.0 (37.3)	25.5 (29.3)
Oak	23.6 (38.5)	19.1 (20.4)

*SPT *skin prick test

**Fig. 2:**
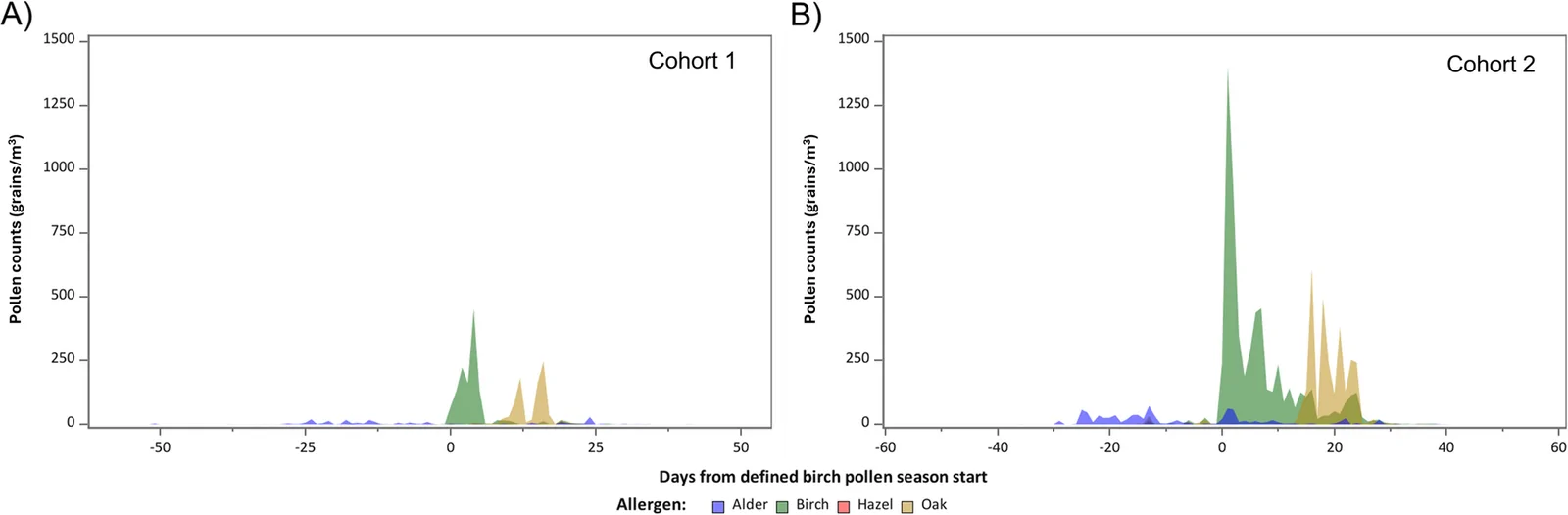
Average tree pollen counts in Canada for the (**A**) 2022 season (cohort 1) and (**B**) 2023 season (cohort 2). Average is from 2 pollen stations in Canada weighted by the number of participants assigned to each station. The graph includes data for days where at least 10 participants in one of the treatment groups filled in the eDiary

Birch pollen counts during the BPS were higher during the 2023 season (cohort 2 enrollment) than the 2022 season (cohort 1 enrollment), at an average (SD) of 240 (12) and 195 (12) grains/m^3^, respectively (Table 2; Fig. 2). Oak pollen counts were also notably higher in the 2023 OPS than the 2022 OPS, at an average of 244 (34) and 90 (16), respectively (Table 2). Alder pollen was below the defined threshold in 2022 and was very low in 2023; hazel pollen was below the defined threshold in both 2022 and 2023 (Table 2).

**Table 2:** Tree pollen counts and season duration in Canada for the 2022 season (cohort 1) and 2023 season (cohort 2)

Pollen	2022 (Cohort 1, *n* = 43)				2023 (Cohort 2, *n* = 44)			
	Average grains/m^3^, (SD)	Median grains/m^3^	Range grains/m^3^	Duration of season, d*	Average grains/m^3^, (SD)	Median grains/m^3^	Range grains/m^3^	Duration of season, d*
Birch	195 (12)	199	151–199	7	240 (12)	243	196–243	32
Alder	0	0	0	0	21 (4)	20	20–36	33
Hazel	0	0	0	0	0	0	0	0
Oak	90 (16)	94	33–94	14	244 (34)	253	121–253	19

*From at least 1 site in season

The duration of the birch and oak pollen seasons in 2022 was 7 and 14 days, respectively (Table 2). The pollen seasons for birch and alder in 2023 lasted approximately 1 month and the oak season lasted for 19 days (Table 2).

## Efficacy

**Fig. 3:**
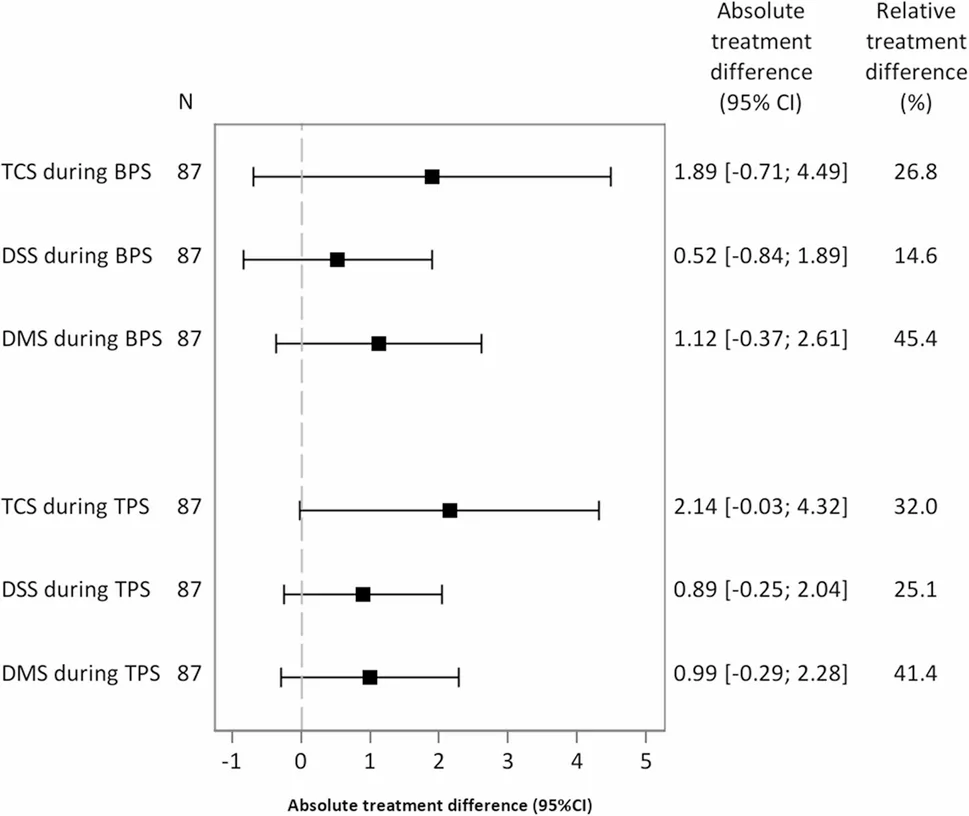
Outcomes in the Canadian subpopulation during BPS and TPS. BPS, birch pollen season; DMS, daily medication score; DSS, daily symptom score; TCS, total combined score; TPS, tree pollen season

**Fig. 4:**
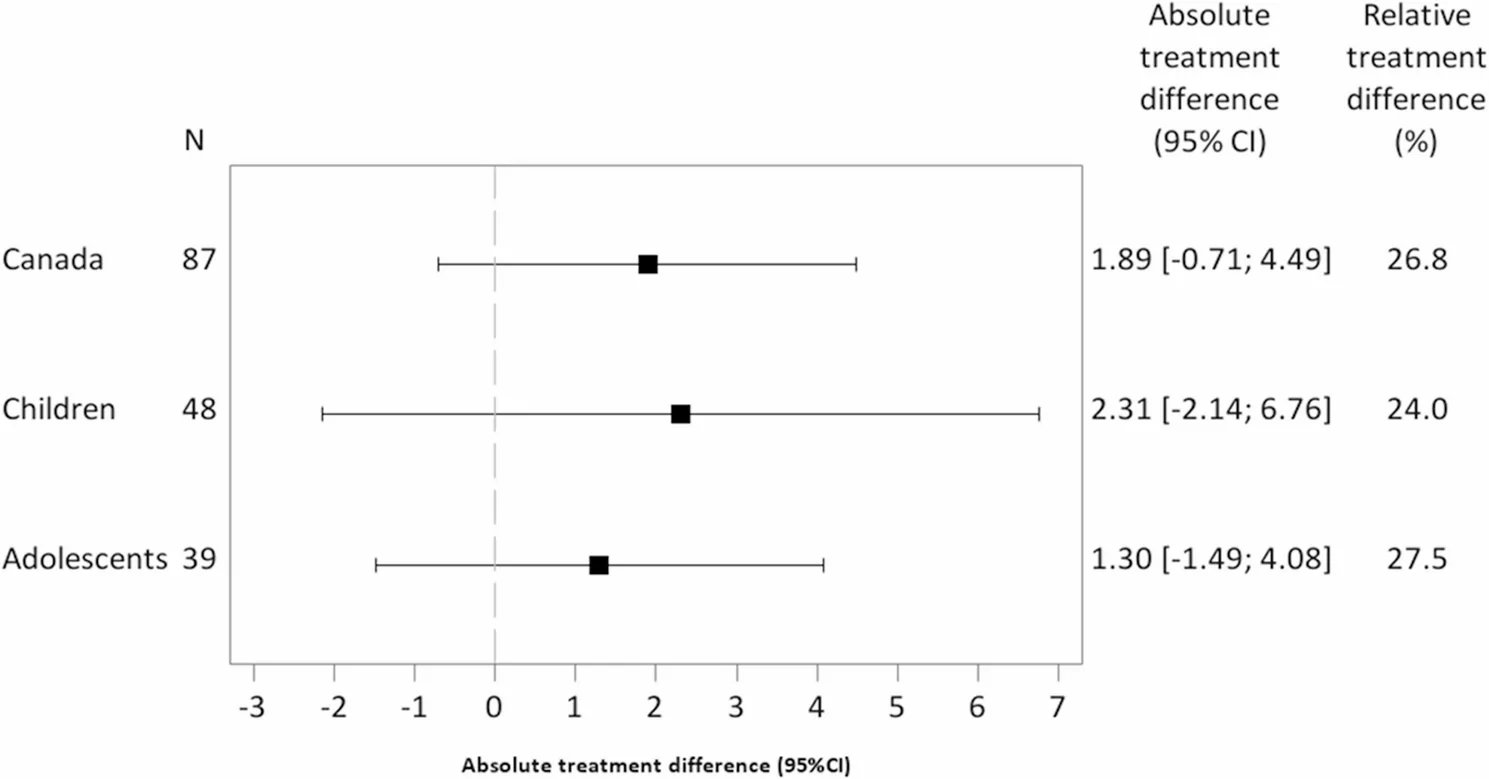
Average TCS during BPS in the Canadian subpopulation by age subgroups. BPS, birch pollen season; TCS, total combined score

In the Canadian subpopulation, treatment with the tree SLIT-tablet reduced the average TCS during BPS by 26.8% versus placebo (absolute difference [95% CI] = 1.89 [− 0.71, 4.49], *p* = 0.138; Fig. 3). During the TPS, the treatment difference in average TCS was statistically significant, with a reduction of 32.0% with tree SLIT-tablet versus placebo (absolute difference = 2.14 [− 0.03, 4.32], *p* = 0.042; Fig. 3). Reductions in DSS and DMS with the tree SLIT-tablet versus placebo were also observed during the BPS and TPS (Fig. 3**)**. For the TCS during BPS, results were consistent among the children (ages 5–11 y) and adolescent (ages 12–17 y) subgroups, with absolute differences that corresponded to a reduction with tree SLIT-tablet versus placebo of 24.0% and 27.5%, respectively (Fig. 4).

In the total trial population, tree SLIT-tablet treatment significantly reduced the average TCS by 19.2% versus placebo during BPS (absolute difference = 1.13 [0.42, 1.84], *p* = 0.002) and 16.8% during TPS (absolute difference = 0.76 [0.26, 1.26], *p* = 0.003).

**Fig. 5:**
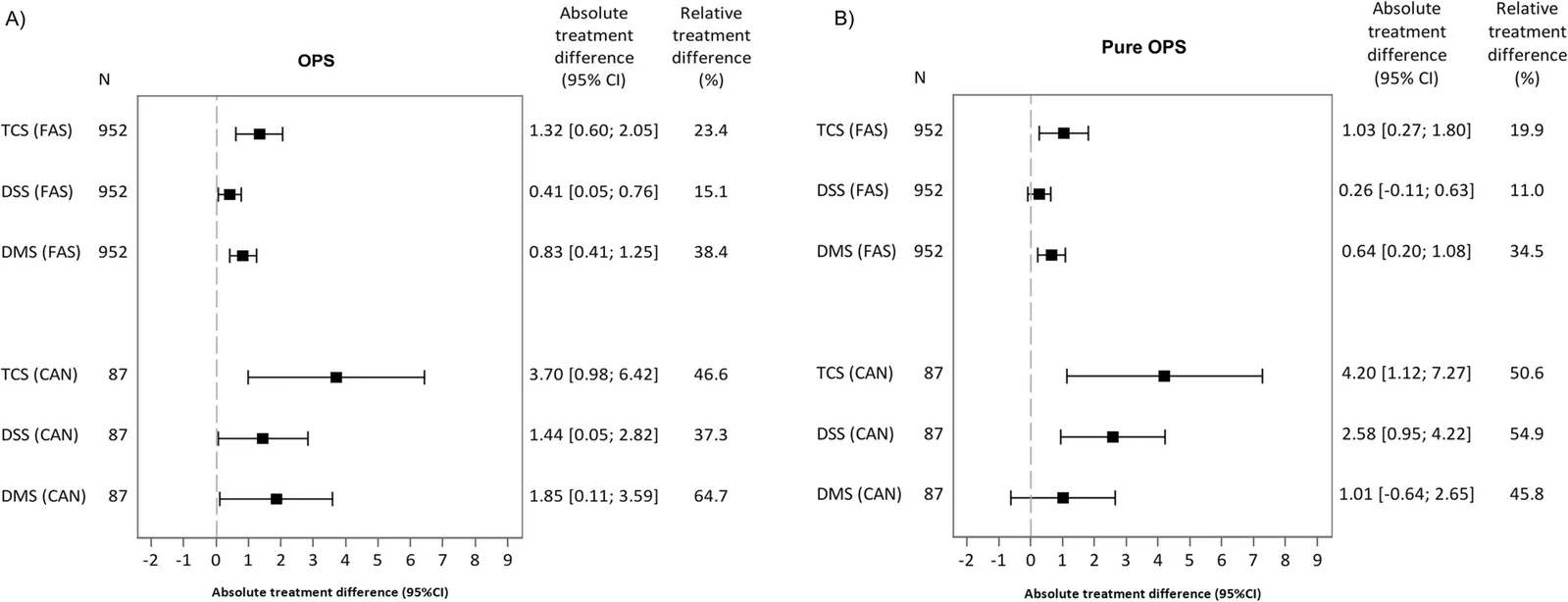
Outcomes in the FAS of the total population and the Canadian subpopulation during (**A**) OPS and (**B**) pure OPS. Pure OPS has no overlap of birch, alder, or hazel pollen seasons. CAN, Canadian subpopulation; DMS, daily medication score; DSS, daily symptom score; FAS, full analysis set; OPS, oak pollen season; TCS, total combined score

In both the Canadian subpopulation and total trial population, treatment differences in outcomes significantly favored the tree SLIT-tablet versus placebo during the OPS and pure OPS (Fig. 5 and Supplemental Table S1). In the Canadian subpopulation, the average TCS with tree SLIT-tablet was reduced by 46.6% versus placebo during OPS (absolute difference = 3.70 [0.98, 6.42), *p* = 0.004). Similar results were observed with the average TCS during pure OPS, with a reduction of 50.6% (absolute difference = 4.20 [1.12, 7.27], *p* = 0.005).

In the total trial population, tree SLIT-tablet treatment reduced the average TCS by 23.4% versus placebo during OPS (absolute difference = 1.32 [0.60, 2.05], *p* < 0.001) and 19.9% during pure OPS (absolute difference = 1.03 [0.27, 1.80], *p* = 0.009).

### Safety

The tree SLIT-tablet was well tolerated in the total trial population and the Canadian subpopulation. In the Canadian subpopulation, there were no treatment-related serious AEs, events requiring epinephrine administrations, systemic allergic reactions, severe local swellings or mouth/throat edema, severe asthma exacerbations, or events of eosinophilic esophagitis (Table 3). A numerically higher proportion of participants in the tree SLIT-tablet group reported oral allergy syndrome (OAS) compared with the placebo group (11% vs. 0%, respectively). The safety profile was similar between children and adolescents (Table 3). The percentage of participants who discontinued treatment due to AEs was low and similar between the tree SLIT-tablet and placebo groups (4% vs. 5%, respectively).

**Table 3:** Treatment-related adverse events in the Canadian subpopulation overall and by age group

Treatment-related adverse events, *n* (%)	Overall (5–17 years)		Children (5–11 years)		Adolescents (12-17 years)	
	Placebo *n* = 42	Tree SLIT-tablet *n* = 45	Placebo *n* = 23	Tree SLIT-tablet *n* = 25	Placebo *n* = 19	Tree SLIT-tablet *n* = 20
Treatment-related AEs	11 (26)	43 (96)	7 (30)	23 (92)	4 (21)	20 (100)
Severity						
Mild	11 (26)	41 (91)	7 (30)	21 (84)	4 (21)	20 (100)
Moderate	2 (5)	10 (22)	1 (4)	8 (32)	1 (5)	2 (10)
Severe	1 (2)	0	1 (4)	0	0	0
Serious AEs	0	0	0	0	0	0
Discontinuation of study drug due to AEs	2 (5)	2 (4)	2 (9)	2 (8)	0	0
Allergic reactions treated with epinephrine	0	0	0	0	0	0
Oral allergy syndrome	0	5 (11)	0	4 (16)	0	1 (5)
Asthma exacerbations	0	0	0	0	0	0
Severe local swellings	0	0	0	0	0	0
Systemic allergic reactions	0	0	0	0	0	0
Eosinophilic esophagitis	0	0	0	0	0	0

*AEs *adverse events, n=number of participants

## Discussion

Regional differences in biomes may affect preseasonal priming of the immune response and pollen amounts, which in turn can impact the magnitude of the treatment effect of allergy immunotherapy. In this Canadian subanalysis, the tree SLIT-tablet was found to improve tree pollen-induced AR/C outcomes versus placebo, consistent with findings in the overall trial population [19]. The treatment effect was consistent for both children and adolescents.

This analysis provides the first evidence of efficacy and safety of the tree SLIT-tablet in Canadian children and adolescents. Although the pivotal phase III trial in adults for the tree SLIT-tablet did not include North American study sites [31], a Canadian phase II, dose-finding, environmental exposure chamber trial in adults demonstrated efficacy and safety; after 24 weeks, the 12 SQ-Bet dose reduced the total symptom score compared with placebo by 25% during birch pollen exposure and 24% during oak pollen exposure (both *p* < 0.05) [10]. 

The relative difference of 26.8% in TCS during BPS exceeded the 20% clinically-relative difference for a total combined symptom and medication score proposed by the World Allergy Organization [32]. A 16% relative improvement from placebo has also been validated as the minimal clinically important difference (MCID) for the EAACI-recommended combined symptom and medication score [33]. Furthermore, in the primary analysis of the full trial population, an absolute difference of at least 1 in the primary endpoint between the tree SLIT-tablet and placebo was considered the MCID that the trial was designed to demonstrate [19]. In the Canadian subpopulation, this MCID was met with an absolute difference in TCS during BPS of 1.89. A 1-point absolute difference in the primary endpoint is considered clinically relevant as it represents a clinically meaningful effect for individual patients. For example, a 1-point reduction in the average TCS during the BPS may correspond to one of the following scenarios:

A difference of 1 obtained entirely from a change in rhinitis symptoms could correspond to a reduction in one severity class (e.g. from ‘severe’ to ‘moderate’) for one symptom every single day during the relevant pollen season. That is, instead of experiencing a rhinitis symptom that is hard to tolerate and causes interference with activities of daily living and/or sleeping every day of the pollen season, the subject experiences this symptom as bothersome but tolerable.

A difference of 1 obtained entirely from a change in rhinitis rescue medication use could correspond to a reduction in antihistamine use of approximately 5 days per month for a patient that previously would have had to take antihistamines on a daily basis to relieve symptoms (a 1-point daily reduction in the antihistamine component of the daily medication score [i.e., 1/6] is equivalent to a 17% reduction in the number of days when antihistamines are used).

The demonstrated significant treatment effect of the tree SLIT-tablet during OPS is highly relevant to Canada, where oak is found throughout much of the southern region of the country [5]. In contrast, pollen counts from hazel and alder, two other trees in the birch homologous group common in Europe, were very low in 2022 and 2023 in the Canadian study sites. In the pivotal phase III adult trial of the tree SLIT-tablet conducted in Europe and Russia, distinct birch, hazel, alder, and oak pollen seasons were recorded [31]. Compared with placebo, the tree SLIT-tablet demonstrated a significant improvement in TCS during the BPS, OPS, and the combined hazel/alder season [31, 34]. In addition, the current analysis found a 51% relative improvement in TCS from placebo during the pure OPS (i.e., eliminating any days that overlapped with birch, hazel, or alder pollen seasons), supporting a highly-effective, specific impact of the tree SLIT-tablet on oak pollen-induced AR/C. Together, these results indicate a consistent effect of the tree SLIT-tablet on AR/C induced by pollen from trees in the birch homologous group. Such consistency supports the suggestion from professional societies to use a single extract representative of an allergen homologous group for allergy immunotherapy in patients who exhibit sensitivity to multiple cross-reactive allergens [35–37]. 

The magnitudes of the absolute and relative differences in TCS between the tree SLIT-tablet and placebo were greater in the Canadian subpopulation than in the full trial population for all the measured pollen seasons (i.e., BPS, TPS, OPS). In a pooled analysis of two adult ragweed SLIT-tablet trials, a greater magnitude difference in TCS during peak pollen season was also observed in Canadian participants s (39.5% vs. placebo) compared with the full population (23.2% vs. placebo) [25]. The appearance of a greater treatment effect of the SLIT-tablets in the Canadian subpopulations may simply be an artifact of the smaller sample sizes, as analyses of multiple phase III trials for SLIT-tablets have demonstrated a consistent treatment effect across geographic regions [38]. Alternatively, a pronounced priming effect related to early alder and high birch pollen exposure in the second cohort may have impacted the high treatment effect observed in the later occurring oak season.

Safety data for the Canadian children and adolescents showed the tree SLIT-tablet was well-tolerated in these populations. A higher percentage of OAS with the tree SLIT-tablet was reported in the Canadian subpopulation compared with the total population (11% vs. < 2%, respectively) [19] and compared with adults (< 5%) [31]. Two of the 5 participants had existing OAS before the trial but reported an AE of OAS during the trial. Regional reporting differences may reflect a heightened emphasis on OAS in Canada, where Canadian investigators may have questioned participants and caregivers about OAS in greater detail.

This analysis is based on the Canadian subgroup that included a small number of participants (*n* = 87), and hence the results should be interpreted with caution. A limitation is that scoring of symptoms and medication use for children was conducted primarily by caregivers, adding a layer of subjectivity to the primary outcome measures. Notably, the shorter duration of the BPS and OPS and relatively low pollen counts during the 2022 season (cohort 1 enrollment) may have reduced the measurable treatment effect of the tree SLIT-tablet. Collectively, these factors contribute to variability in the outcome measures reported by individual participants, especially pronounced in subgroup analyses. Despite these limitations, the efficacy results in the Canadian subgroup consistently favored the tree SLIT-tablet.

## Conclusions

Results of this subanalysis of Canadian children and adolescents participating in a large, phase III, placebo-controlled trial demonstrate that the tree SLIT-tablet has favorable efficacy during both the BPS and OPS and is well tolerated in this population. The consistent efficacy and favorable safety profile observed in this Canadian subpopulation support the inclusion of the tree SLIT-tablet as a therapeutic option in Canadian clinical practice, broadening the choices for allergy immunotherapy in the pediatric population. Since birch and oak pollen are common triggers of AR/C in Canada, the tree SLIT-tablet offers a convenient and promising treatment option for Canadian children and adolescents with tree pollen-induced AR/C through multiple tree pollen seasons.

## Supplementary Information

Supplementary Material 1.

## Data Availability

All data generated during this study are included within this published article (and its supplementary information files).

## Abbreviations

AE: Adverse event
AIT: Allergen immunotherapy
AR/C: Allergic rhinitis and/or conjunctivitis
BPS: Birch pollen season
DMS: Daily medication score
DSS: Daily symptom score
OAS: Oral allergy syndrome
OPS: Oak pollen season
SLIT: Sublingual immunotherapy
TCS: Total combined score
TPS: Tree pollen season
