# Supplementary material for: Efficacy and safety of the tree sublingual immunotherapy tablet in the subpopulation of Canadian children with allergic rhinitis and/or conjunctivitis: phase III trial results
Source: Allergy Asthma Clin Immunol. 2026 Jul 11;22:55. doi: 10.1186/s13223-026-01054-w (PMC13640251; doi:10.1186/s13223-026-01054-w)
Supplement: Supplementary file 1 [file 13223_2026_1054_MOESM1_ESM.docx]

**Supplemental Material**

**Figure S1.** Trial design. Reproduced under Creative Commons Attribution-NonCommercial-NoDerivs License from Gappa M, et al, 2024.[1] BPS, birch pollen season; EOT, end of treatment; SLIT, sublingual immunotherapy; TC, telephone contact; TPS, tree pollen season; V, visit.

**Table S1.** TCS during the OPS and pure OPS in the total trial population and Canadian subpopulation.

|  | **Treatment Group** | **No. Observed (No. Imputed)** | **TCS Adjusted Mean** | **Absolute Difference, placebo-tree SLIT-tablet (95% CI)** | **Percentage Relative to Placebo (95% CI)** | **P Value** |
| --- | --- | --- | --- | --- | --- | --- |
| **OPS** |  |  |  |  |  |  |
| Total trial population | Placebo | 445 (34) | 5.67 |  |  |  |
|  | Tree SLIT-tablet | 443 (30) | 4.34 | 1.32 (0.60, 2.05) | 23.4 (11.4, 33.9) | <0.001 |
| Canadian subpopulation | Placebo | 40 (2) | 7.94 |  |  |  |
|  | Tree SLIT-tablet | 45 (0) | 4.24 | 3.70 (0.98, 6.42) | 46.6 (18.4, 69.1) | 0.004 |
| **Pure OPS** |  |  |  |  |  |  |
| Total trial population | Placebo | 415 (64) | 5.20 |  |  |  |
|  | Tree SLIT-tablet | 418 (55) | 4.16 | 1.03 (0.27, 1.80) | 19.9 (5.5, 32.3) | 0.009 |
| Canadian subpopulation | Placebo | 20 (22) | 8.30 |  |  |  |
|  | Tree SLIT-tablet | 23 (22) | 4.10 | 4.20 (1.12, 7.27) | 50.6 (21.3, 75.2) | 0.005 |

OPS, oak pollen season; SLIT, sublingual immunotherapy; TCS, total combined score.

**References**

1. Gappa M, Gagnon R, Horak F, Cichocka-Jarosz E, Dalgaard T, Hargreaves K, et al. The SQ tree sublingual immunotherapy tablet is effective and well tolerated in children-A pivotal phase III trial. Allergy. 2025;80:795-806.
